# Supplementary material for: Multi-functional 2D hybrid aerogels for gas absorption applications
Source: Sci Rep. 2021 Jun 30;11:13548. doi: 10.1038/s41598-021-92957-8 (PMC8245581; doi:10.1038/s41598-021-92957-8)
Supplement: Supplementary file 1 — Supplementary Information. [file 41598_2021_92957_MOESM1_ESM.docx]

*Supporting Information*

**Multi-functional 2D hybrid aerogels for gas absorption applications**

Charalampos Androulidakis^1^, Maria Kotsidi^1,2^, George Gorgolis^1^, Christos Pavlou^1,2^, Labrini Sygellou^1^, George Paterakis^1,2^, Nick Koutroumanis^1,2^ and Costas Galiotis^1,2*^

^1^Institute of Chemical Engineering Sciences, Foundation of Research and Technology-Hellas (FORTH/ICE-HT), Stadiou Street, Platani, Patras, 26504 Greece

^2^Department of Chemical Engineering, University of Patras, Patras 26504 Greece

^*^Corresponding author: [c.galiotis@iceht.forth.gr](mailto:c.galiotis@iceht.forth.gr) ; galiotis@chemeng.upatras.gr

1. **Literature survey for VOC absorption of graphene-based materials**

**Table S1**: Comparative table on gas absorption of other rGO-based composite materials and the hybrid aerogels (with the hBN platelets addition) of this work

| **Sample** | **Maximum adsorption capacity (gg^-1^)** | **Reference** |
| --- | --- | --- |
| Freeze-dried rGO | 0.276 (benzene)  0.304 (toluene) | [1] |
| Hierarchically Porous Graphene/ZIF-8 Hybrid Aerogel | 0.03 (carbon dioxide) | [2] |
| Amine-functionalized Macroscopic Graphene Aerogel | 0.002 (formaldehyde) | [3] |
| rGO aerogel | 0.94 (carbon dioxide) | [4] |
| 3D Amino-Functional Graphene-Sponge Composites Decorated  by Graphene Nanodots | 0.022 (formaldehyde) | [5] |
| Reduced Graphene Oxide/Polymer Monolithic  Materials | 0.135 – 0.146 (carbon dioxide) | [6] |
| Polypyrrole/  reduced graphene oxide highly porous material | 0.663 (benzene)  0.258 (carbon dioxide) | [7] |
| Reduced Graphene Oxide with microwave treatment and KOH (rGOMWKOH) | 0.001 (acetaldehyde) | [8] |
| MIL-101/Graphene Oxide | 2.368 (CCL_4_, carbon tetrachloride) | [9] |
| ZIF-8/Graphene Oxide | 0.240 (CH_2_Cl_2_, dichloromethane) | [10] |
| Amino-functional Graphene Aerogel | 0.274 (formaldehyde) | [11] |
| **This work** | **24 (formaldehyde)**  **4.04 (hydrochloric acid)** | **-** |

The absorption capacity of similar materials that is found in the literature [1-11] are listed in Table S1. Our results show orders of magnitude higher absorbance albeit under somewhat different experimental conditions. In this work, the adsorbed capacity (gg^-1^) of the hybrid rGO-based aerogels was calculated by:

$$Q= \frac{m_{1}- m_{0}}{m_{0}}$$

where *m_0_* and *m_1_* are the weights of the aerogel before and after gas/VOC absorption, respectively. The *m_1_* value is the weight of the material for the maximum uptake of the measured gas. This measurement method is based on the gravimetric difference of the aerogels and does not include any specially-designed apparatus for examining the absorption capacity of the aerogels [1-11]. The samples were then placed in a desiccator and exposed to saturated gas controlled environment, while their mass weight gain due to absorption was monitored by regular gravimetric measurements until the maximum mass increment was reached. On the other hand, in published works [1-11], the VOC is originated from a gas mixture system which usually uses the tested gas mixed with nitrogen or oxygen in order to achieve the desired flow rate and concentration (ppm or ppb). Nevertheless, following our method, we were able to detect the selectivity of the as-prepared hybrid aerogels since different absorption capacities were obtained for different material (% hBN platelets addition) and different tested VOC. The issue of the sensitivity of these aerogels has to be further examined with an analogous device that will integrate some VOCs sensors and record the absorption of the hybrid aerogels for known gas concentrations.

**2. Structural integrity of the aerogels under gas flow.**

Initially, two samples, one of neat rGO and one of rGO/hBN 0.5/0.5 ratio were examined and loaded in a cylindrical cell (tube) made of PMMA, as shown in Figure S1a and b. The two edges of the cell were open for the studied gas stream to have the ability to pass through the cell. The studied gas that was chosen is nitrogen, which is one of the most common inert gases. Also, a flowmeter was connected to the supplier of nitrogen in order to show the gas flow which passes through the experimental cell (Figure S1c). The flowmeter could measure the gas flow in L/min. The PMMA cylindrical cell had a length equal to 12.8 cm and an inner diameter equal to 1.85 cm.

For proving that no decomposition of the material is obtained during and after a gas flow, the weight of the examined samples was initially measured, and then for specific time intervals after being blown with nitrogen. A weight meter of high accuracy was exploited which is placed exactly next to the experimental setup. The used flow that was studied is 2 L/ min, while the time intervals were 10, 40 and finally 100 mins. As shown in Table S2, it was found that no significant weight loss is obtained after 10, 40 and 100 mins. A significant weight loss would indicate that the material has been mechanically decomposed and consequently been released into the atmosphere. Both the gravimetric measurements and the optical observation do not lead us to such a conclusion. It is worth-mentioning that after 100 minutes, for both samples, the recorded mass is slightly higher possibly due to nitrogen adsorption.

Table S2: Table of measured masses for the examined samples, after blowing them with a flow equal to 2 L/min

| **Examined sample** | **Starting mass**  **(mg)** | **Mass after 10 minutes (mg)** | **Mass after 40 minutes (mg)** | **Mass after 100 minutes (mg)** |
| --- | --- | --- | --- | --- |
| rGO | 51.0 | 50.9 | 50.6 | 52.1 |
| rGO/hBN | 17.4 | 18.2 | 18.3 | 18.4 |


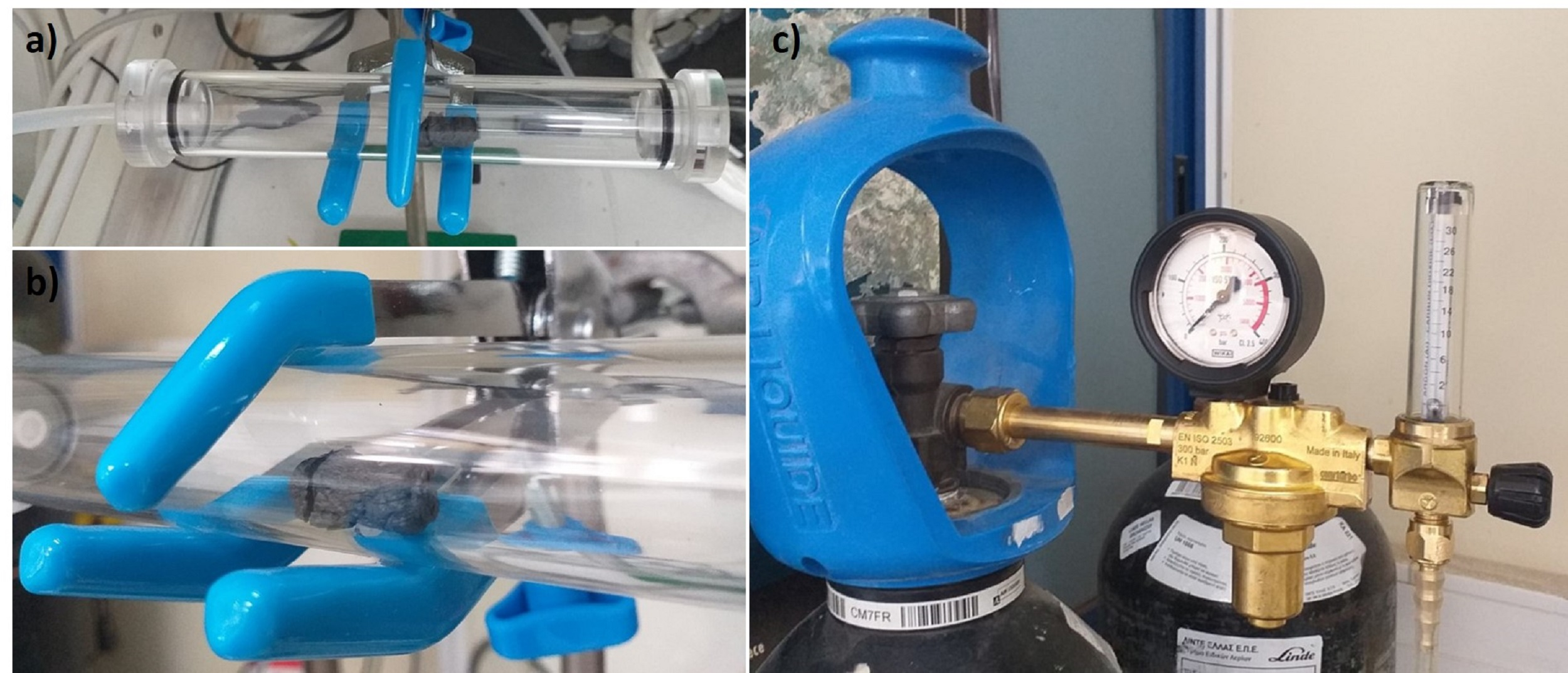


**Figure S1:** a) The cylindrical cell with the examined aerogel inside it during the gas flow test. The aerogel was mounted in the middle of the cell, b) the cell from a closer view, and c) the gas flowmeter that was used, was connected to the nitrogen gas cylinder.

**3. EDS spectra of the rGO and rGO-hBN aerogels.**

In this section we present EDS measurements performed on the rGO and the hybrid rGO-hBN aerogels. In figure S2 (a) and (b), the SEM images of the rGO and rGO-hBN aerogels are presented. The corresponding EDS spectra for the rGO and rGO-hBN are given in (c) and (d), respectively. The EDS spectra measured based on the area denoted by the green frame in (a), (b). For the hybrid aerogel the spectrum clearly shows the presence of B, N atoms, further supporting the Raman and XRD measurements.


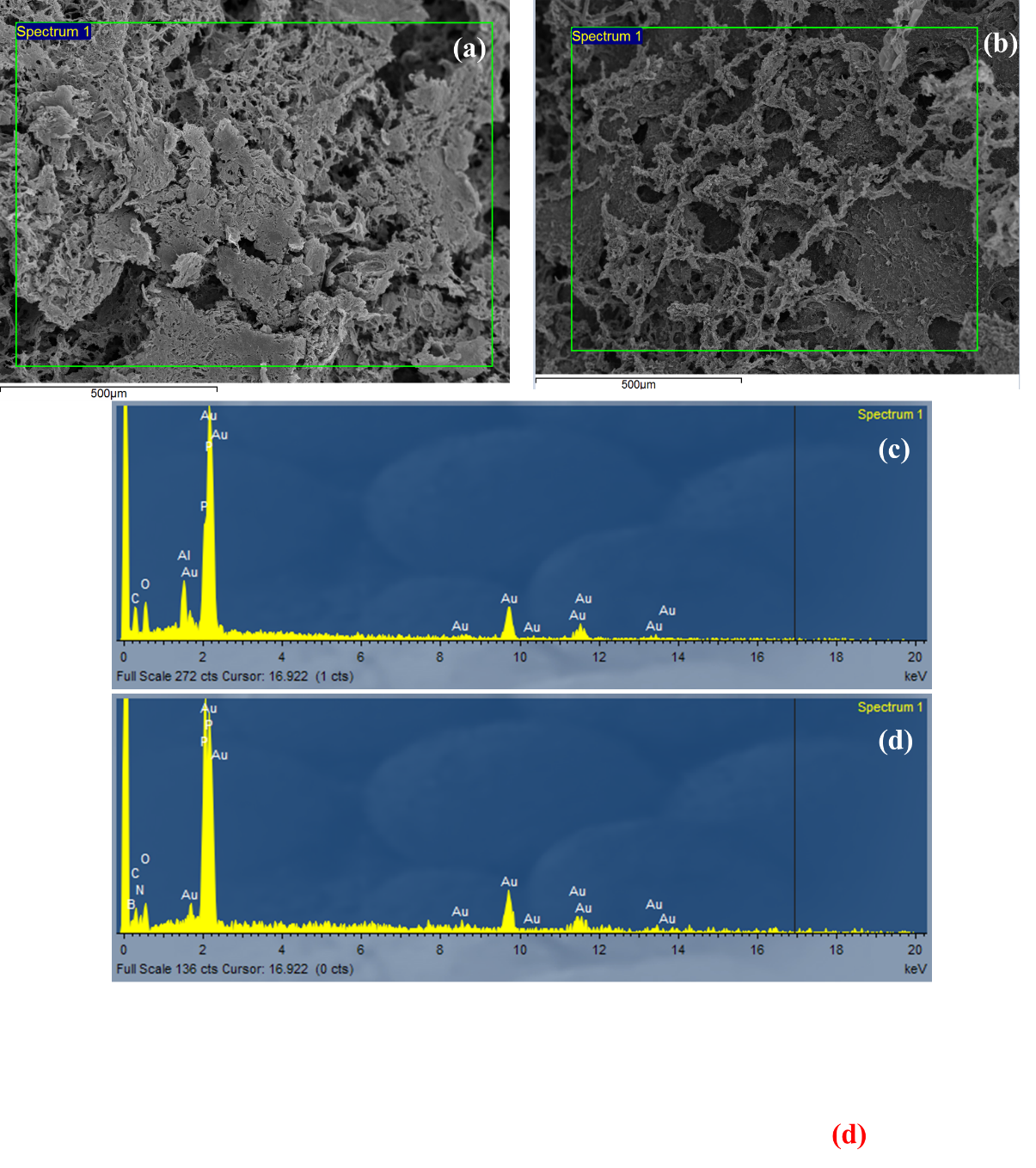


**Figure S2**. SEM images of (a) rGO and (b) rGO-hBN aerogels. The green frame represents the area from which the EDS spectra were measured. The corresponding EDS spectra are given in (c) for the rGO and (d) for the rGO-hBN aerogels, respectively.

**4. N_2_ adsorption-desorption experiments for hybrid aerogels.**

Specific surface area and porosity measurements were performed by obtaining the volumetric nitrogen adsorption at 78 K, using an “*autosorb iQ Model 7*” gas sorption system and high purity gases (>99.999%). Before the measurement, the hybrid aerogel rGO/hBN 50/50 was outgassed at 150°C under vacuum (P < 10^−5^ mbar) for 10 hours. The specific surface area was found following the multi-point BET equation. The pore volume was calculated from the adsorbed nitrogen at 0.90 relative pressure. The pore size distribution was determined with the Barrett-Joyner-Halenda (BJH) model.

First of all, the specific surface area (SSA) of the hybrid rGO/hBN 50/50 aerogel that was examined herein, was calculated using the analysis software of the gas sorption system and found to be equal to 18 m^2^/g. This value is relatively low for a graphene aerogel [12] but has been obtained in other studies for similar hybrid materials [13-15]. Also, as already explained, the hBN platelets that have been incorporated in the aerogel structure do increase the density of such materials, which in turn results in the decrease of the measured specific surface area. The nitrogen adsorption-desorption isotherms, actually, are more useful for porosities up to 50 nm or a little more, and for this range, the as-prepared hybrid aerogel exhibits low porosity. Certainly, the majority of the existing pores within the aerogel is in the range of micrometers, as already shown in the corresponding SEM images. Based on the shape of the rGO/hBN nitrogen adsorption-desorption isotherm (**Figure S3a**), we conclude that the material shows a Type II isotherm with a Type H3 hysteresis loop, according to IUPAC classification [16]. Such loops are given by non-rigid aggregates of plate-like particles [16] but also if the pore network consists of macropores which are not completely filled with pore condensates. This finding is in agreement with the SEM measurements which show clearly the existence of a macropores network. Additionally, from the pore size distribution of **Figure S3b**, we can deduce that the hybrid aerogel is characterized by both micro-porosity (<2 nm) and meso-porosity (between 2 and 50 nm). The pore volume that was found from the adsorbed N_2_ for a 0.90 relative pressure is equal to 0.015.


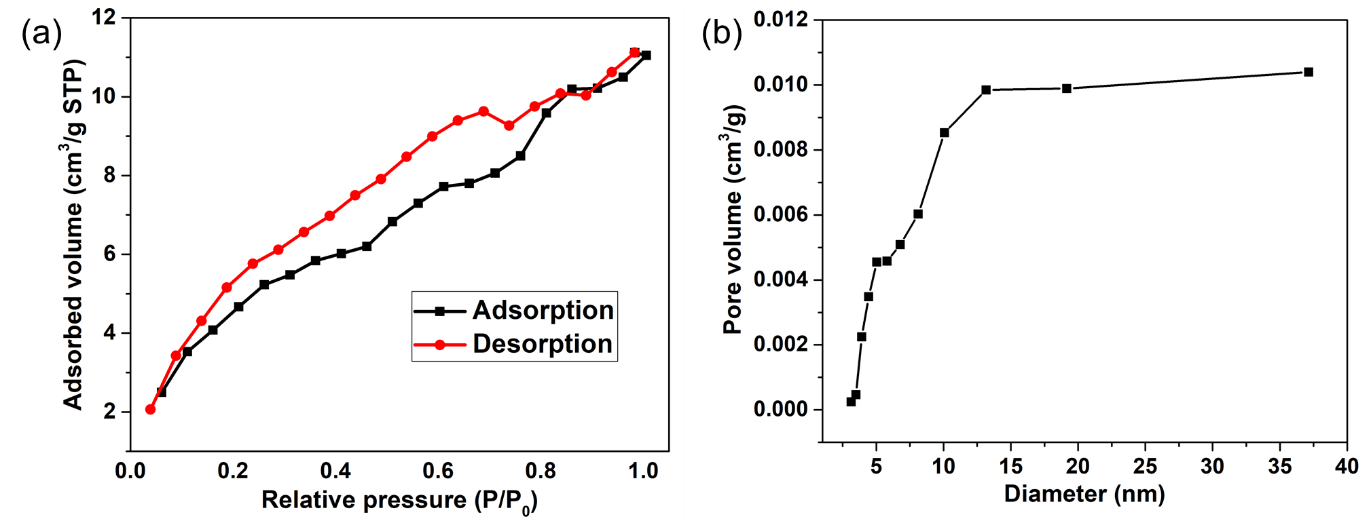


**Figure S3**. (a) Pore characterization of the rGO/hBN 50/50 hybrid aerogel. The nitrogen adsorption–desorption isotherm of the aerogel is shown. The BET surface area and nitrogen adsorption–desorption isotherms were measured at using the analysis program of the micropore physio-/chemisorption analyzer. (b) Barrett-Joyner-Halenda (BJH) pore size distribution curve of the examined aerogel.

**REFERENCES**

[1] L. Yu, L. Wang, W. Xu, L. Chen, M. Fu, J. Wu, D.J.J.o.E.S. Ye, 67 (2018) 171-178.

[2] M. Jiang, H. Li, L. Zhou, R. Xing, J.J.A.a.m. Zhang, interfaces, 10 (2018) 827-834.

[3] J. Liang, Z. Cai, L. Li, L. Guo, J.J.R.A. Geng, 4 (2014) 4843-4847.

[4] R. Sevanthi, F. Irin, D. Parviz, W.A. Jackson, M.J.J.R.a. Green, 6 (2016) 43401-43407.

[5] L. Wu, L. Zhang, T. Meng, F. Yu, J. Chen, J.J.A. Ma, A.Q. Research, 15 (2015) 1028-1034.

[6] N. Politakos, I. Barbarin, T. Cordero-Lanzac, A. Gonzalez, R. Zangi, R.J.P. Tomovska, 12 (2020) 936.

[7] B. Szczęśniak, Ł. Osuchowski, J. Choma, M.J.J.o.P.M. Jaroniec, 25 (2018) 621-627.

[8] J.M. Kim, J.H. Kim, C.Y. Lee, D.W. Jerng, H.S.J.J.o.h.m. Ahn, 344 (2018) 458-465.

[9] Y. Zheng, F. Chu, B. Zhang, J. Yan, Y.J.M. Chen, M. Materials, 263 (2018) 71-76.

[10] Y. Zhou, L. Zhou, X. Zhang, Y.J.M. Chen, M. Materials, 225 (2016) 488-493.

[11] L. Wu, Z. Qin, L. Zhang, T. Meng, F. Yu, J.J.N.J.o.C. Ma, 41 (2017) 2527-2533.

[12] J.-Y. Hong, E.-H. Sohn, S. Park, H.S.J.C.E.J. Park, 269 (2015) 229-235.

[13] Y.-X. Ma, X. Li, W.-J. Shao, Y.-L. Kou, H.-P. Yang, D.-J.J.J.o.n. Zhang, nanotechnology, 20 (2020) 2205-2213.

[14] J. Xie, J. Li, L. Zhao, X. Zhang, B. Yu, R. Wu, R. Wang, J.-H. Liu, F. Xue, S.-T.J.N. Yang, N. Letters, 6 (2014) 1018-1023.

[15] S.M. Seraji, X. Jin, Z. Yi, C. Feng, N.V.J.N.R. Salim, (2021) 1-10.

[16] M. Thommes, K. Kaneko, A.V. Neimark, J.P. Olivier, F. Rodriguez-Reinoso, J. Rouquerol, K.S.J.P. Sing, a. chemistry, 87 (2015) 1051-1069.
